# Investigating the causal impact of gut microbiota on trigeminal neuralgia: a bidirectional Mendelian randomization study

**DOI:** 10.3389/fmicb.2025.1420978

**Published:** 2025-02-27

**Authors:** Chuan Zeng, Chaolong Zhang, Yuxuan Jia, Huaiyu Zhou, Chunming He, Haimin Song

**Affiliations:** ^1^The First Clinical Medical College of Gannan Medical University, Ganzhou, Jiangxi, China; ^2^Department of Neurosurgery, First Affiliated Hospital of Gannan Medical University, Ganzhou, Jiangxi, China

**Keywords:** trigeminal neuralgia, gut microbiota, Mendelian randomization, causality, gut-brain axis

## Abstract

**Background:**

The etiology and pathogenesis of trigeminal neuralgia remain unclear. This study examines the connection between gut microbiota and trigeminal neuralgia using Mendelian randomization analysis to provide insights into the disorder’s origin and propose potential therapies based on our findings.

**Methods:**

We used data from the MiBioGen consortium (13,266 participants) for gut microbiota and the IEU OpenGWAS project (800 cases, 195,047 controls) for trigeminal neuralgia. We checked for heterogeneity and horizontal pleiotropy and used the inverse variance weighting method as our main approach to study the causal link between gut bacteria and trigeminal neuralgia, MR-Egger, simple mode, weighted median, and weighted mode as supplementary methods, with a sensitivity test using leave-one-out analysis. If a bacteria-trigeminal neuralgia link was found, we conducted a reverse analysis for confirmation.

**Results:**

According to the final results, these groups include *Butyricimonas* (Genus, id = 945, *p*-value = 0.007, OR = 1.742, 95% CI: 1.165–2.604), *unknowngenus* (Genus, id = 1000005479, *p*-value = 0.005, OR = 1.774, 95% CI: 1.187–2.651) and *Bacteroidales* (Family, *p*-value = 0.005, OR = 1.774, 95% CI: 1.187–2.651) were causally associated with trigeminal neuralgia. No significant results according to reverse Mendelian randomization analysis.

**Conclusion:**

In our study, we identified specific gut bacteria linked to trigeminal neuralgia. To comprehensively understand their impact and mechanisms, additional randomized trials are necessary.

## Introduction

Trigeminal neuralgia (TN) presents as recurring, paroxysmal bursts of intense pain within the trigeminal distribution area, devoid of any discernible impairment of trigeminal nerve functionality (Cruccu et al., 2016). This affliction predominantly affects females and tends to emerge more frequently in individuals around the age of 40 (Maarbjerg et al., 2017). Diagnosis hinges upon three pivotal criteria: the manifestation of pain localized within one or more branches of the trigeminal nerve, abrupt and excruciating paroxysms of pain often likened to “shocks” or “electric jolts,” lasting a mere fraction of a minute—typically seconds (Bendtsen et al., 2020; Cruccu et al., 2020). The etiology and pathogenesis of TN remain shrouded in ambiguity. Current elucidation posits that the compression of the trigeminal nerve by ectopic, contorted blood vessels within the pontine bridge along the posterior root may instigate localized demyelination, thereby precipitating the onset of painful episodes (Love and Coakham, 2001). Pathological alterations in TN chiefly unveil through the detection of vacuoles within the cytoplasm of trigeminal ganglion cells, along with irregular proliferation, hypertrophy, distortion, or even vanishing of axons. Furthermore, discernible thickening and disintegration of myelin sheaths, coupled with segmental demyelinating changes in the majority of fibers, constitute primary pathological hallmarks of this condition (Love and Coakham, 2001).

In recent investigations, the discerning gaze of scientific inquiry has illuminated the potential influence wielded by metabolites intricately linked to the gut microbiota upon diverse facets of inflammation and immune response (Foster and McVey Neufeld, 2013). Furthermore, the burgeoning body of research accentuates the emergence of a bidirectional conduit for communication between the encephalon and the gastrointestinal realm, heralded as the gut-brain axis (Lyu et al., 2022). This revelation ignites a tantalizing inquiry: might the gut microbiota exert a discernible sway over the pathogenesis and evolution of trigeminal neuralgia? Delving into this provocative query unfurls novel pathways for comprehending the labyrinthine interplay between gut microbiota and TN, thereby furnishing profound insights into the intricate machinations underpinning the inception and progression of this neurological malady. Nevertheless, the definitive establishment of a correlation between gut flora and trigeminal neuralgia remains ensconced in uncertainty, primarily attributed to the paucity of evidence stemming from randomized controlled trials. While randomized controlled trials stand as the pinnacle for corroborating correlation in epidemiological inquiries, their execution is often hindered by ethical constraints and formidable financial burdens. In order to reveal the potential correlation between gut microbiota and trigeminal neuralgia, we explored the causal relationship between gut flora and trigeminal neuralgia by Mendelian Randomization (MR), on the basis of which we hypothesized the correlation between the two (Porcu et al., 2019; Saunders et al., 2022). Mendelian Randomization ingeniously employs genetic variability as an instrumental tool to simulate interventions, thereby augmenting our ability to make more robust inferences concerning the impact of a factor on the incidence of the disease. In the forthcoming investigation, we will wield MR methodologies to scrutinize the putative causal interrelation between gut microbiota and TN.

The paramount aim of this investigation is to delve into the conceivable correlation existing between the intricate makeup of the gut microbiota and the susceptibility to TN, with a deliberate emphasis on unveiling the cryptic biological underpinnings. We envisage that the findings gleaned from this scholarly pursuit will furnish a trove of innovative perspectives and methodologies for the prospective mitigation and management of TN. Beyond the mere elucidation of TN’s etiological landscape, this ambitious endeavor harbors the potential to furnish substantial scaffolding for the formulation of tailored therapeutic regimens, thereby heralding the advent of enhanced well-being and ameliorated quality of life for individuals grappling with this neurological affliction.

## Material and methods

### Study design

**FIGURE 1:**
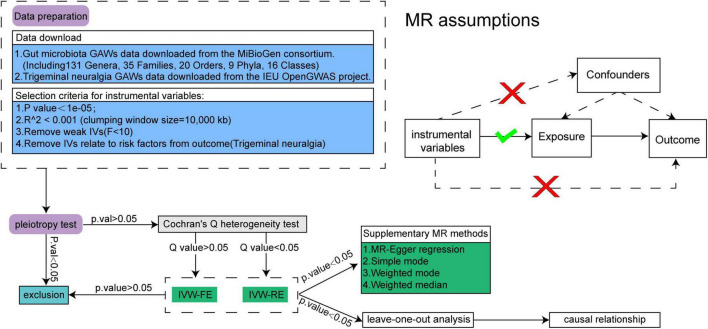
The whole study design.

The intricate framework of our comprehensive study design is elegantly depicted in Figure 1. Our methodological rigor was exemplified through the application of Mendelian Randomization (MR), meticulously employed to scrutinize the causal interplay between the gut microbiota and TN. Adherence to the triad of foundational tenets governing MR analysis was paramount: (1) Establishing a robust nexus between genetic variability and exposure factors; (2) Ensuring the independence of genetic variability from confounding variables (Jia et al., 2023); (3) Validating that genetic variability exerts its sole influence on outcomes via exposure factors, while eschewing involvement in alternative pathways (Jin et al., 2023). Concurrently, we undertook a reverse MR analysis, leveraging the statistically significant revelations from the initial MR inquiry to fortify and refine our conclusions.

We procured the succinct summary statistics delineating the composition of the gut microbiota from the preeminent genome-wide meta-analysis conducted to date, meticulously orchestrated by the esteemed MiBioGen consortium (van der Velde et al., 2019).^1^ This exhaustive analysis encompassed a cohort of 18,340 individuals of European descent hailing from 11 nations, encapsulating an expansive dataset comprising 122,110 loci of genetic variability. Concomitantly, the pertinent summary statistics germane to the genome-wide association study (GWAS) for TN were acquired from the esteemed bastion of the Medical Research Council Integrative Epidemiology Unit (IEU) Open GWAS project [Trait: Trigeminal neuralgia - IEU OpenGWAS project (mrcieu.ac.uk)] (IEU Open GWAS project, n.d.), updated to the illustrious epoch of 2021.04.06. The TN cohort comprised 800 cases from European descent juxtaposed against 195,047 controls, with a comprehensive amalgam of 16,380,408 single nucleotide polymorphisms (SNPs) delineating the genetic landscape.

The meticulous curation of instrumental variables (IVs) was governed by a systematic protocol, meticulously delineated through the following methodological steps: (1) Initial Identification: Single nucleotide polymorphisms (SNPs) demonstrating association with each genus at the locus-wide significance threshold (*P* < 1.0 × 10−5) were earmarked as prospective IV candidates (Liu et al., 2022; Zeng et al., 2023). (2) Linkage Disequilibrium Analysis: Following the preliminary selection, a rigorous examination of linkage disequilibrium (LD) was undertaken for all IVs. A stringent threshold of r2 < 0.001 was imposed, with a clumping window size of 10,000 kb, ensuring the independence of selected IVs. If the number of instrumental variables was less than 10, the bacterial flora was excluded. (3) Relevance Assessment: SNPs linked to the exposure but lacking corresponding matches within the genome-wide association study outcome statistics were judiciously pruned. This elimination process, guided by the formula F = beta^^^2_exposure / SE^^^2_exposure, was instrumental in guaranteeing the pertinence of the selected instrumental variables to both the exposure and outcome in the subsequent analysis (Jin et al., 2023). (4) Allele Frequency Threshold: SNPs characterized by a minor allele frequency (MAF) ≤ 0.01 were discerningly excluded from further consideration, ensuring the robustness of subsequent analyses. (5) Resolution of Palindromic SNPs: In instances involving palindromic SNPs, meticulous determination of forward strand alleles was meticulously executed based on allele frequency information. This meticulous step aimed to foster consistency and lucidity in the portrayal of genetic information throughout the analytical process (Li et al., 2022).

### Statistical analysis

In the quest to unravel potential causal links between bacterial taxa and trigeminal neuralgia, the venerable tool of Mendelian Randomization (MR) was deftly wielded. Prior to commencing our analytical voyage, a meticulous scrutiny for horizontal pleiotropy was undertaken, aimed at identifying and preemptively excluding statistics susceptible to the confounding influence of horizontal pleiotropic effects. This preemptive maneuver, undertaken with sagacious forethought, served as a bulwark fortifying the integrity of our analysis, thereby endowing the inverse variance weighting (IVW) method with the mantle of primacy in adjudicating causality within the realm of MR analysis (Burgess et al., 2016). Moreover, the discerning eye of scrutiny was cast upon the landscape of heterogeneity among instrumental variables, courtesy of Cochrane’s Q test. Detection of heterogeneity (*P* < 0.05) prompted the adoption of a judicious recourse to the random-effects IVW (IVW-RE) model, renowned for its penchant in furnishing conservative estimates. Conversely, in instances where heterogeneity lay dormant, a steadfast embrace of the fixed-effects IVW (IVW-FE) model ensued, deemed apt for scenarios characterized by the steadfast consistency of instrumental variable effects. This adaptive approach, attuned to the ebbs and flows of heterogeneity among the selected instrumental variables, epitomized a hallmark of methodological finesse (Zeng et al., 2023). Subsequent to the unveiling of statistically significant results from the IVW analysis (*p* < 0.05), a veritable arsenal of additional MR methodologies was marshaled forth, including MR-Egger regression, simple mode, weighted median, and weighted mode. Noteworthy is the selection of weighted median and MR-Egger regression, undertaken to supplement the IVW method, thereby imbuing our analyses with a wider expanse of confidence intervals (CIs). These supplementary forays into the analytical landscape were conceived with the noble intent of buttressing the resilience of our findings and delving into the nuanced tapestry of the causal nexus between the gut microbiota and trigeminal neuralgia (Slob and Burgess, 2020).

In a final stride towards bolstering the veracity of our findings, we subjected the p-values corresponding to statistically significant causality to a meticulous leave-one-out analysis, culminating in resolute outcomes. Subsequent to this, in an earnest bid to fortify the edifice of our conclusions, we embarked upon an inverse Mendelian Randomization (MR) analysis. In this undertaking, we leveraged Genome-Wide Association Study summary statistics stemming from flora causally intertwined with trigeminal neuralgia as the outcome, juxtaposed against those emanating from TN as the exposure. Notably, the arsenal of analytical tools was deftly wielded within the realm of the R programming language, complemented by the versatile “TwoSampleMR” package, thereby furnishing a robust platform for methodical exploration and inference. This methodological rigor, underpinned by the synergistic interplay between analytical precision and computational prowess, lent an aura of credibility and gravitas to our investigative odyssey (Hemani et al., 2017; Hemani et al., 2018).

## Results

**FIGURE 2:**
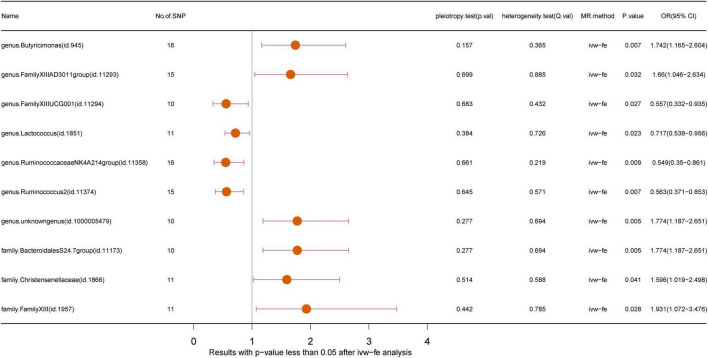
Forest plot of GM taxa associated with GBM (*P* < 0.05) identified by IVW-FE method.

**FIGURE 3:**
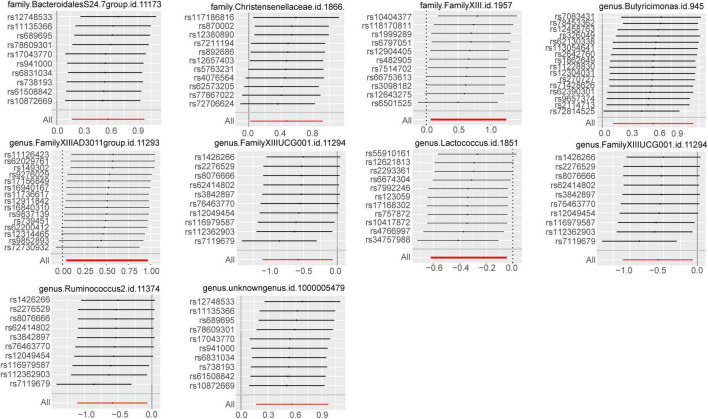
Leave-one-out plots for the causal association between gut microbiota and GBM identified by IVW-FE method.

**FIGURE 4:**
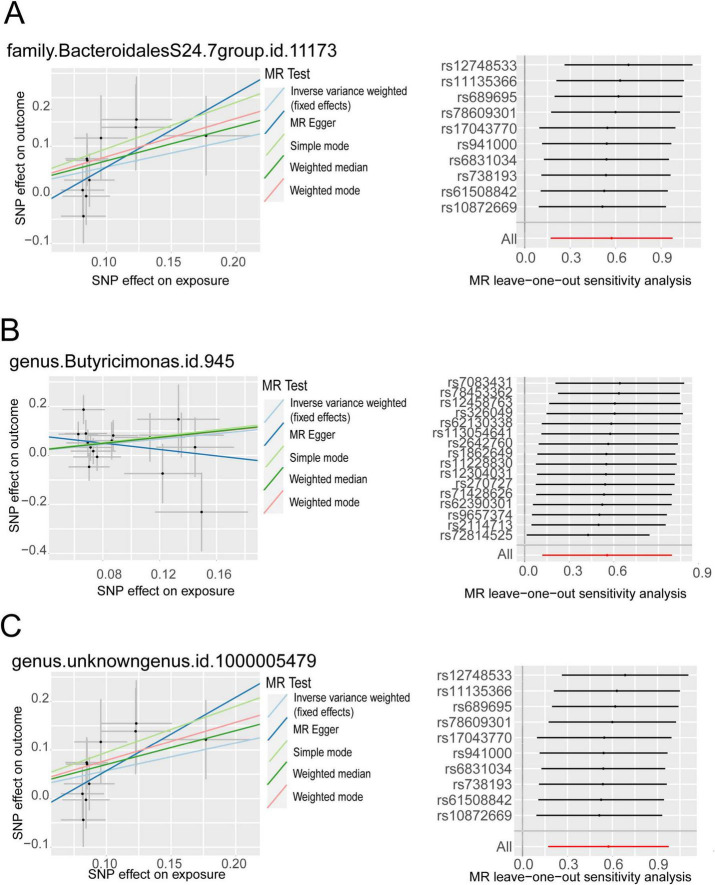
The MR positive results screened after the leave-one-out analysis. **(A)** Scatter plot of MR results for *BacteroidalesS24.7group* on the left, Leave-one-out analysis results on the right; **(B)** Scatter plot of MR results for Butyricimonas on the left, Leave-one-out analysis results on the right; **(C)** Scatter plot of MR results for unknown genus (id.1000005479) on the left, Leave-one-out analysis results on the right.

We harnessed the comprehensive gut microbiota Genome-Wide Association Study data procured from the esteemed MiBioGen consortium, encompassing a rich tapestry of taxonomic resolution. Within this expansive dataset, a total of 131 genus-level taxa, 35 family-level taxa, 20 order-level taxa, 9 phylum-level taxa, and 16 class-level taxa were meticulously curated, affording a granular insight into the multifaceted landscape of microbial composition. This breadth of taxonomic resolution facilitated a nuanced exploration of potential associations between microbial taxa and trigeminal neuralgia, enriching our analytical endeavors with depth and granularity. Following the outlined methodology, we meticulously executed a horizontal pleiotropy test to discern and exclude statistics potentially influenced by horizontal pleiotropy. Subsequently, we engaged diverse Inverse Variance Weighting (IVW) analysis methods contingent upon the Q-value derived from the heterogeneity test. Remarkably, the Q-value for the heterogeneity test surpassed the threshold of 0.05 across nearly all cohorts, indicative of a notable absence of statistical heterogeneity. As delineated in Figure 2, Supplementary Figure 1, and Supplementary Table 1, the IVW analysis method unveiled associations between trigeminal neuralgia and 7 genus-level flora [*Butyricimonas, FamilyXIIIAD3011group, FamilyXIIIUC, Lactococcus, RuminococcaceaeNK4A214group, Ruminococcus2, unknowngenus*(id.1000005479)] alongside 3 family-level flora (*BacteroidalesS24.7group, Christensenellaceae, FamilyXIII*). However, discerning from the results of the leave-one-out analysis (Figure 3), we prudently excluded seven flora exhibiting sensitivity. The conclusive outcomes of the forward Mendelian Randomization analysis were meticulously tabulated in Table 1 and illustrated in Figure 4. Notably, *Butyricimonas* (Genus, IVW, *P* = 0.007, OR = 1.742, 95%CI:1.165–2.604; Weighted median, *P* = 0.030, OR = 1.847, 95% CI: 1.063–3.211), *unknowngenus(id.1000005479)* (Genus, IVW, *P* = 0.005, OR = 1.774, 95%CI:1.187–2.651; Weighted median, *P* = 0.011, OR = 2.014, 95% CI: 1.172–3.462), and *BacteroidalesS24.7group* (Family, IVW, *P* = 0.005, OR = 1.774, 95%CI:1.187–2.651; Weighted median, *P* = 0.014, OR = 2.014, 95% CI: 1.152–3.524) were substantiated in two MR methods to harbor causality with TN (IVW and weighted median). Furthermore, *BacteroidalesS24.7group* (Family, IVW, *P* = 0.005, OR = 1.774, 95%CI: 1.187–2.651; Weighted median, *P* = 0.014, OR = 2.014, 95% CI: 1.152–3.524; Simple mode, *P* = 0.049, OR = 2.587, 95% CI: 1.142–5.861) evinced causality with TN across three distinct methods (IVW, Simple mode, and weighted median). Detailed information regarding Single Nucleotide Polymorphisms (SNPs) can be found in Supplementary Table 2.

**TABLE 1:** The results of MR analysis.

Name (id)	No. of SNP	Pleiotropy test (*p*-value)	Cochrane’s Q heterogeneity test (Q_pval)	MR method	*p*-val	OR	or_lci95	or_uci95
genus.Butyricimonas (id.945)	16	0.157	0.365	ivw-fe	0.007	1.742	1.165	2.604
				MR Egger	0.447	0.520	0.101	2.674
				Simple mode	0.167	1.919	0.796	4.629
				Weighted mode	0.156	1.826	0.828	4.026
				Weighted median	0.030	1.847	1.063	3.211
genus.unknowngenus (id.1000005479)	10	0.277	0.694	ivw-fe	0.005	1.774	1.187	2.651
				MR Egger	0.107	4.546	0.888	23.277
				Simple mode	0.064	2.587	1.071	6.248
				Weighted mode	0.080	2.194	1.006	4.782
				Weighted median	0.011	2.014	1.172	3.462
family.BacteroidalesS24.7 group(id.11173)	10	0.277	0.694	ivw-fe	0.005	1.774	1.187	2.651
				MR Egger	0.107	4.546	0.888	23.277
				Simple mode	0.049	2.587	1.142	5.861
				Weighted mode	0.081	2.194	1.001	4.806
				Weighted median	0.014	2.014	1.152	3.524

No. of SNP, number of SNPs being used as IVs.; ivw-fe, fixed-effects inverse variance weighting; OR, Odds Ratio; or_lci95-or_uci95, 95% confidence interval; Significant *P*-value was marked in red.

Employing the taxa delineated above, we embarked upon a reverse MR analysis, with the Genome-Wide Association Study data of bacteria enlisted as the exposure. Elaborate details pertaining to the single nucleotide polymorphisms (SNPs) deployed as instrumental variables are meticulously cataloged in Table 2. Regrettably, the outcomes of the reverse MR analysis failed to unveil any significant results, thereby corroborating the notion that trigeminal neuralgia may not exert a discernible impact on the abundance of intestinal flora. This alignment with the concept of a functional disorder of trigeminal neuralgia serves to further bolster the credibility and robustness of our findings, reinforcing the reliability and validity of our investigative endeavors.

**TABLE 2:** The detail information of SNPs in trigeminal neuralgia.

SNP	Effect_allele	Other_allele	beta	se	eaf	pval
rs72640584	G	A	0.6285	0.1363	0.03858	3.97E−06
rs116501213	G	A	1.0847	0.2398	0.01381	6.10E−06
rs141103938	A	G	1.9571	0.4319	0.005103	5.85E−06
rs6531785	G	A	−0.7635	0.1614	0.9713	2.24E−06
rs79054949	G	A	0.5838	0.1231	0.04856	2.09E−06
rs10214062	C	T	0.3263	0.0726	0.1448	7.07E−06
rs75568875	G	A	2.2983	0.5177	0.003709	9.03E−06
rs9767181	T	C	0.4203	0.0938	0.08363	7.39E−06
rs62501575	A	T	0.2555	0.0566	0.2958	6.41E−06
rs79459523	G	A	5.0421	1.0424	0.0011	1.32E−06
rs9550562	A	G	0.2491	0.0548	0.3122	5.57E−06
rs111365054	T	C	0.678	0.1443	0.03512	2.61E−06
rs148953513	C	A	1.0163	0.2285	0.01533	8.71E−06
rs3810268	T	C	0.2572	0.0518	0.3826	6.88E−07
rs182954926	C	A	3.3932	0.7661	0.00196	9.46E−06
rs13052134	A	G	0.7143	0.1554	0.02998	4.29E−06

SNP, single nucleotide polymorphism; SE, standard error.

## Discussion

In the pursuit of unraveling the intricate relationship between gut microbiota and Trigeminal Neuralgia, our paramount objective was to employ a Mendelian Randomization analysis imbued with methodological rigor. To this end, we judiciously harnessed the aggregated gut microbiota statistics gleaned from the extensive Genome-Wide Association Study meta-analysis meticulously orchestrated by the MiBioGen consortium. Concurrently, we complemented this robust dataset with aggregated TN statistics sourced from the IEU OpenGWAS project release data, thus ensuring the foundation of our study rested upon a bedrock of empirical validity.

Within this analytical crucible, our discerning gaze alighted upon three specific microbial taxa: *Butyricimonas*, an *unknown genus (id.1000005479)*, and the *Bacteroidales S24.7 group*, each exhibiting notable causality with Trigeminal Neuralgia (TN). Intriguingly, the results of our exhaustive analysis appear to suggest that *Butyricimonas, the unknown genus (id.1000005479)*, and *the Bacteroidales S24.7 group* may act as harbingers of increased risk for the development of trigeminal neuralgia. However, given the enigmatic nature of the unknown genus, our subsequent discussion will pivot upon the implications stemming from the findings related to *Butyricimonas* and *the Bacteroidales S24.7 group.*

The genus *Butyricimonas*, renowned for its production of butyrate, a pivotal energy source for colonic cells, occupies a prominent position in the pantheon of gut microbiota. Butyrate, primarily metabolized into energy by colonic cells, serves as a linchpin in promoting gut health, fostering the release of host-derived antimicrobial peptides (AMPs), and stimulating the secretion of immunoglobulin A (IgA). These activities collectively orchestrate a potent anti-inflammatory immune response, thereby conferring protection against allergen-induced airway issues, ameliorating airway hyperresponsiveness, and thwarting the development of intestinal tumors, as evidenced in murine models.

Recent investigations have underscored the indispensable role of *Butyricimonas* in preserving central nervous system (CNS) homeostasis, orchestrating inflammation modulation, and assuaging neuropathic pain (Linnerbauer et al., 2020; Stoeva et al., 2021). Despite being lauded as a probiotic owing to its multifaceted anti-inflammatory, anti-tumor, and immune-boosting properties, the intriguing findings stemming from our Mendelian Randomization analysis suggest a potential paradox. Contrary to prevailing notions, our analysis posits *Butyricimonas* as a putative risk factor for trigeminal neuralgia, thereby challenging conventional wisdom and underscoring the complexity inherent in the gut-brain axis and its implications for neurological disorders.

Building upon Lin et al. (2021)’s seminal work, which delineated a trigeminal neuralgia mouse model and elucidated the role of infiltrating macrophages and lymphocytes in chronic compression nerve injury, we extrapolate further insights into the pathogenesis of this debilitating condition. It is postulated that these immune cells traverse the compromised blood-nerve barrier under chronic compression, guided by specific chemokines, thereby orchestrating the onset and progression of trigeminal neuralgia. In light of these observations, we propose a novel hypothesis implicating *Butyricimonas* and its metabolites in the etiology of trigeminal neuralgia. It is conjectured that *Butyricimonas*-induced alterations in trigeminal ganglion cells manifest as vacuoles in the plasma, irregular proliferation, hypertrophy, distortion, or even disappearance of axons, alongside marked thickening and disintegration of myelin sheaths, and segmental demyelinating changes in the majority of fibers. These structural aberrations may precipitate the onset and progression of trigeminal neuralgia by fostering the release of host-derived antimicrobial peptides (AMPs) or secretory immunoglobulin A (IgA), thus perpetuating a cascade of inflammatory events culminating in neuronal dysfunction and pain. This hypothesis presents a tantalizing avenue for further investigation, shedding light on the intricate interplay between gut microbiota, immune response, and neurological pathology in trigeminal neuralgia.

*Bacteroidales*, an order of bacteria nestled within the Bacteroidetes phylum, represents a cadre of Gram-negative organisms that find ubiquity across diverse ecosystems, including the intricate milieu of the human gut. This taxonomic order harbors a mosaic of bacterial species, each endowed with a repertoire of functions, ranging from beneficial to pivotal in the digestion of complex carbohydrates within the gut milieu. Eckburg et al. (2005)’s observational study stands as a testament to the multifaceted role played by Bacteroidales metabolites, unveiling their profound anti-inflammatory effects. Furthermore, Hu et al. (2020)’s seminal work sheds light on the potential therapeutic implications of Bacteroidales S24.7 in mitigating inflammation within the context of inflammatory bowel disease (IBD). These seminal findings underscore the nuanced interplay between gut microbiota, inflammation, and disease pathogenesis, painting a picture of Bacteroidales as a linchpin in maintaining gut homeostasis and orchestrating immunomodulatory responses (Hu et al., 2020).

The landscape surrounding the Bacteroidales S24.7 group within the family Bacteroidales unfolds with complexity and contradiction, as elucidated by Ormerod et al. (2016)’s report. Their findings shed light on the dual nature of this microbial cohort, capable of both triggering infections by inciting immune responses and synthesizing virulence factors. Furthermore, another Mendelian Randomization analysis, delving into the causal nexus between periodontitis and gut microbiota, lends credence to the notion that the family Bacteroidales S24.7 group might indeed exacerbate the risk of periodontitis (Luo et al., 2023). The discordance observed across these studies underscores the intricacies inherent in the gut microbiome and its multifaceted interactions with inflammatory diseases. Individual variations in gut microbiological composition, coupled with the intricate interplay of multifactorial properties characterizing inflammatory conditions, contribute to this tapestry of inconsistency. In light of these nuances, the MR analysis, grounded upon the assumptions delineated in Figure 1, endeavors to navigate through this labyrinth of complexity. Within this analytical framework, we proffer a conjecture positing that the *Bacteroidales S24.7 group* and its metabolites may indeed play a contributory role in the onset and progression of trigeminal neuralgia. By stimulating the production of inflammatory responses, this microbial cohort potentially orchestrates a cascade of events culminating in neuronal dysfunction and pain. However, further elucidation through rigorous investigation is imperative to unravel the true mechanistic underpinnings of this phenomenon.

Rooted in the conceptual framework of the gut-brain axis elucidated in prior research, this study stands at the vanguard of scientific inquiry, poised to unravel the intricate interplay between the human gut microbiota and the central nervous system (CNS). Through the conduit of the gut-brain axis, the human gut microbiota exerts a profound influence on the development and function of the CNS, underscoring its pivotal role in shaping neurological processes (Dehhaghi et al., 2020). This study boasts several methodological advantages, chief among them being the utilization of Mendelian Randomization (MR) analysis—a powerful tool for establishing causal relationships by harnessing existing genetic variations in nature. Through the mechanism of randomization simulation, MR analysis affords the unique ability to mimic assignment to a control group, thereby furnishing researchers with heightened confidence in formulating causal hypotheses. By employing genetic variation as instrumental variables, MR analysis effectively circumvents issues stemming from confounding and reverse causation, thereby facilitating a clearer elucidation of relationships between variables (Hong et al., 2023). In contrast to observational studies fraught with myriad limitations such as confounding, selection bias, and memory bias, Mendelian Randomization analysis offers a robust alternative. By leveraging genetic variation gleaned from the most comprehensive meta-analysis of global genomic studies, this study ensures the availability of robust instrumental variables for MR analysis. Through this meticulously crafted approach, causal relationships between gut microbiota and Trigeminal Neuralgia are unearthed, thereby attenuating confounding factors and ameliorating concerns pertaining to the reversal of causality in causal inference (Birney, 2022). Furthermore, the employment of a two-sample MR design, leveraging non-overlapping exposure and outcome pooled data, serves to further bolster the integrity of the study design, mitigating bias and enhancing the robustness of the findings. In summation, this study represents a paradigm shift in our understanding of the intricate relationship between gut microbiota and Trigeminal Neuralgia, offering invaluable insights into the pathophysiological mechanisms underpinning this neurological disorder (Long et al., 2023).

While this study employs a rigorous methodology, there are several important caveats to consider. First, the limited number of Single Nucleotide Polymorphisms (SNPs) analyzed under the conventional Genome-Wide Association Study (GWAS) significance threshold (*P* < 5 × 10^^^−8) required adjustments to enhance sensitivity analysis and reduce potential effects of horizontal pleiotropy. This adjustment underscores the complex relationship between statistical thresholds and the reliability of findings in genetic research.

Additionally, the inherent limitations of Mendelian Randomization (MR) analysis, such as vulnerability to demographic and genetic sequencing errors, should be acknowledged. Given that our study population is of European ancestry, the generalizability of our findings to other demographic groups may be limited, emphasizing the need for careful interpretation and contextualization of results within specific population frameworks.

And it is worth mentioning that in our quest for trigeminal neuralgia GWAS data, we were unable to find data typed according to the Eller/Burchiel protocol (Eller et al., 2005). Moreover, the data utilized in this study originated from Finnish databases of trigeminal neuralgia GWAS data, where rigorous typing was lacking (The description of the data can be found here), thus emphasizing a limitation of our study.

Lastly, the classification of *Butyricimonas* and the *Bacteroidales S24.7 group* as probiotics underscores the multifaceted nature of their roles in gut health. Altering the abundance of these flora, albeit potentially beneficial in alleviating TN symptoms, may engender unintended consequences. Therefore, in the development of TN treatment programs, careful consideration of the broader implications of modulating gut microbiota is imperative to ensure holistic and personalized therapeutic interventions.

## Conclusion

In this two-sample Mendelian Randomization study, we identified several gut microbiota species with causal associations to trigeminal neuralgia. Particularly significant was the causal relationship observed with *Butyricimonas* and *Bacteroidales S24.7 group*, suggesting potential implications for trigeminal neuralgia treatment. To comprehensively understand the negative role of *Butyricimonas* and *Bacteroidales S24.7 group* in trigeminal neuralgia and unveil its precise mechanisms, additional randomized controlled trials are necessary.

## Data Availability

Publicly available datasets were analyzed in this study. This data can be found here: the esteemed MiBioGen consortium (https://mibiogen.gcc.rug.nl); GWAS for Trigeminal neuralgia (https://gwas.mrcieu.ac.uk/datasets/finn-b-G6_TRINEU/).

## Footnotes

1
https://mibiogen.gcc.rug.nl

## References

[B1] Bendtsen L. Zakrzewska J. M. Heinskou T. B. Hodaie M. Leal P. R. L. Nurmikko T. (2020). Advances in diagnosis, classification, pathophysiology, and management of trigeminal neuralgia. *Lancet Neurol.* 19 784–796. 10.1016/S1474-4422(20)30233-7 32822636

[B2] Birney E. (2022). Mendelian randomization. *Cold Spring Harb. Perspect. Med.* 12:a041302. 10.1101/cshperspect.a041302 34872952 PMC9121891

[B3] Burgess S. Dudbridge F. Thompson S. G. (2016). Combining information on multiple instrumental variables in Mendelian randomization: Comparison of allele score and summarized data methods. *Stat. Med.* 35 1880–1906. 10.1002/sim.6835 26661904 PMC4832315

[B4] Cruccu G. Di Stefano G. Truini A. (2020). Trigeminal neuralgia. *N. Engl. J. Med.* 383 754–762. 10.1056/NEJMra1914484 32813951

[B5] Cruccu G. Finnerup N. B. Jensen T. S. Scholz J. Sindou M. Svensson P. (2016). Trigeminal neuralgia: New classification and diagnostic grading for practice and research. *Neurology* 87 220–228. 10.1212/WNL.0000000000002840 27306631 PMC4940067

[B6] Dehhaghi M. Kazemi Shariat, Panahi H. Heng B. Guillemin G. J. (2020). The gut microbiota, kynurenine pathway, and immune system interaction in the development of brain cancer. *Front. Cell. Dev. Biol.* 8:562812. 10.3389/fcell.2020.562812 33330446 PMC7710763

[B7] Eckburg P. Bik E. Bernstein C. Purdom E. Dethlefsen L. Sargent M. (2005). Diversity of the human intestinal microbial flora. *Science (New York, N.Y.)* 308 1635–1638. 10.1126/science.1110591 15831718 PMC1395357

[B8] Eller J. L. Raslan A. M. Burchiel K. J. (2005). Trigeminal neuralgia: Definition and classification. *Neurosurg. Focus* 18:E3. 10.3171/foc.2005.18.5.4 15913279

[B9] Foster J. A. McVey Neufeld K.-A. (2013). Gut-brain axis: How the microbiome influences anxiety and depression. *Trends Neurosci.* 36 305–312. 10.1016/j.tins.2013.01.005 23384445

[B10] Hemani G. Tilling K. Davey Smith G. (2017). Orienting the causal relationship between imprecisely measured traits using GWAS summary data. *PLoS Genet.* 13:e1007081. 10.1371/journal.pgen.1007081 29149188 PMC5711033

[B11] Hemani G. Zheng J. Elsworth B. Wade K. H. Haberland V. Baird D. (2018). The MR-Base platform supports systematic causal inference across the human phenome. *eLife* 7:e34408. 10.7554/eLife.34408 29846171 PMC5976434

[B12] Hong W. Huang G. Wang D. Xu Y. Qiu J. Pei B. (2023). Gut microbiome causal impacts on the prognosis of breast cancer: A Mendelian randomization study. *BMC Genomics* 24:497. 10.1186/s12864-023-09608-7 37644405 PMC10464481

[B13] Hu L. Jin L. Xia D. Zhang Q. Ma L. Zheng H. (2020). Nitrate ameliorates dextran sodium sulfate-induced colitis by regulating the homeostasis of the intestinal microbiota. *Free Radical Biol. Med.* 152 609–621. 10.1016/j.freeradbiomed.2019.12.002 31811920

[B14] IEU Open GWAS project. *Trigeminal Neuralgia Dataset: Finn-b-G6_TRINEU.*.

[B15] Jia Y. Yao P. Li J. Wei X. Liu X. Wu H. (2023). Causal associations of Sjögren’s syndrome with cancers: A two-sample Mendelian randomization study. *Arthritis Res. Ther.* 25:171. 10.1186/s13075-023-03157-w 37715206 PMC10503000

[B16] Jin Q. Ren F. Dai D. Sun N. Qian Y. Song P. (2023). The causality between intestinal flora and allergic diseases: Insights from a bi-directional two-sample Mendelian randomization analysis. *Front. Immunol.* 14:1121273. 10.3389/fimmu.2023.1121273 36969260 PMC10033526

[B17] Li P. Wang H. Guo L. Gou X. Chen G. Lin D. (2022). Association between gut microbiota and preeclampsia-eclampsia: A two-sample Mendelian randomization study. *BMC Med.* 20:443. 10.1186/s12916-022-02657-x 36380372 PMC9667679

[B18] Lin J. Zhou L. Luo Z. Adam M. I. Zhao L. Wang F. (2021). Flow cytometry analysis of immune and glial cells in a trigeminal neuralgia rat model. *Sci. Rep.* 11:23569. 10.1038/s41598-021-02911-x 34876649 PMC8651642

[B19] Linnerbauer M. Wheeler M. A. Quintana F. J. (2020). Astrocyte crosstalk in CNS inflammation. *Neuron* 108 608–622. 10.1016/j.neuron.2020.08.012 32898475 PMC7704785

[B20] Liu K. Zou J. Fan H. Hu H. You Z. (2022). Causal effects of gut microbiota on diabetic retinopathy: A Mendelian randomization study. *Front. Immunol.* 13:930318. 10.3389/fimmu.2022.930318 36159877 PMC9496187

[B21] Long Y. Tang L. Zhou Y. Zhao S. Zhu H. (2023). Causal relationship between gut microbiota and cancers: A two-sample Mendelian randomisation study. *BMC Med.* 21:66. 10.1186/s12916-023-02761-6 36810112 PMC9945666

[B22] Love S. Coakham H. B. (2001). Trigeminal neuralgia: Pathology and pathogenesis. *Brain* 124 2347–2360. 10.1093/brain/124.12.2347 11701590

[B23] Luo S. Li W. Li Q. Zhang M. Wang X. Wu S. (2023). Causal effects of gut microbiota on the risk of periodontitis: A two-sample Mendelian randomization study. *Front. Cell. Infect. Microbiol.* 13:1160993. 10.3389/fcimb.2023.1160993 37305424 PMC10248501

[B24] Lyu Y. Yang H. Chen L. (2022). Metabolic regulation on the immune environment of glioma through gut microbiota. *Semin. Cancer Biol.* 86 990–997. 10.1016/j.semcancer.2021.05.005 33971263

[B25] Maarbjerg S. Di Stefano G. Bendtsen L. Cruccu G. (2017). Trigeminal neuralgia - Diagnosis and treatment. *Cephalalgia* 37 648–657. 10.1177/0333102416687280 28076964

[B26] Ormerod K. Wood D. Lachner N. Gellatly S. Daly J. Parsons J. (2016). Genomic characterization of the uncultured Bacteroidales family S24-7 inhabiting the guts of homeothermic animals. *Microbiome* 4:36. 10.1186/s40168-016-0181-2 27388460 PMC4936053

[B27] Porcu E. Rüeger S. Lepik K. Santoni F. A. Reymond A. Kutalik Z. (2019). Mendelian randomization integrating GWAS and eQTL data reveals genetic determinants of complex and clinical traits. *Nat. Commun.* 10:3300. 10.1038/s41467-019-10936-0 31341166 PMC6656778

[B28] Saunders C. N. Kinnersley B. Culliford R. Cornish A. J. Law P. J. Houlston R. S. (2022). Relationship between genetically determined telomere length and glioma risk. *Neuro Oncol.* 24 171–181. 10.1093/neuonc/noab208 34477880 PMC8804896

[B29] Slob E. A. W. Burgess S. (2020). A comparison of robust Mendelian randomization methods using summary data. *Genet. Epidemiol.* 44 313–329. 10.1002/gepi.22295 32249995 PMC7317850

[B30] Stoeva M. K. Garcia-So J. Justice N. Myers J. Tyagi S. Nemchek M. (2021). Butyrate-producing human gut symbiont, Clostridium butyricum, and its role in health and disease. *Gut Microbes* 13 1–28. 10.1080/19490976.2021.1907272 33874858 PMC8078720

[B31] van der Velde K. J. Imhann F. Charbon B. Pang C. van Enckevort D. Slofstra M. (2019). MOLGENIS research: Advanced bioinformatics data software for non-bioinformaticians. *Bioinformatics* 35 1076–1078. 10.1093/bioinformatics/bty742 30165396 PMC6419911

[B32] Zeng Y. Cao S. Yang H. (2023). Roles of gut microbiome in epilepsy risk: A Mendelian randomization study. *Front. Microbiol.* 14:1115014. 10.3389/fmicb.2023.1115014 36922970 PMC10010438

