# Supplementary material for: Investigating the causal impact of gut microbiota on trigeminal neuralgia: a bidirectional Mendelian randomization study
Source: Front Microbiol. 2025 Feb 27;16:1420978. doi: 10.3389/fmicb.2025.1420978 (PMC11905160; doi:10.3389/fmicb.2025.1420978)
Supplement: Supplementary file 1 [file Table_1.docx]

**Supplementary Table1.The results of MR analysis.**

| Name (id) | No.of SNP | pleiotropy test (p.value) | Cochrane's Q heterogeneity test(Q_pval) | MR method | p.val | OR | or_lci95 | or_uci95 |
| --- | --- | --- | --- | --- | --- | --- | --- | --- |
| genus.Butyricimonas(id.945) | 16 | 0.157 | 0.365 | ivw-fe | 0.007 | 1.742 | 1.165 | 2.604 |
|  |  |  |  | MR Egger | 0.447 | 0.520 | 0.101 | 2.674 |
|  |  |  |  | Simple mode | 0.167 | 1.919 | 0.796 | 4.629 |
|  |  |  |  | Weighted mode | 0.156 | 1.826 | 0.828 | 4.026 |
|  |  |  |  | Weighted median | 0.030 | 1.847 | 1.063 | 3.211 |
| genus.FamilyXIIIAD3011group(id.11293) | 15 | 0.699 | 0.885 | ivw-fe | 0.032 | 1.660 | 1.046 | 2.634 |
|  |  |  |  | MR Egger | 0.960 | 1.060 | 0.110 | 10.245 |
|  |  |  |  | Simple mode | 0.480 | 1.487 | 0.510 | 4.340 |
|  |  |  |  | Weighted mode | 0.382 | 1.587 | 0.582 | 4.325 |
|  |  |  |  | Weighted median | 0.132 | 1.615 | 0.866 | 3.013 |
| genus.FamilyXIIIUCG001(id.11294) | 10 | 0.683 | 0.432 | ivw-fe | 0.027 | 0.557 | 0.332 | 0.935 |
|  |  |  |  | MR Egger | 0.810 | 0.800 | 0.138 | 4.644 |
|  |  |  |  | Simple mode | 0.081 | 0.351 | 0.124 | 0.999 |
|  |  |  |  | Weighted mode | 0.099 | 0.368 | 0.127 | 1.068 |
|  |  |  |  | Weighted median | 0.016 | 0.421 | 0.208 | 0.851 |
| genus.Lactococcus(id.1851) | 11 | 0.384 | 0.726 | ivw-fe | 0.023 | 0.717 | 0.538 | 0.956 |
|  |  |  |  | MR Egger | 0.210 | 0.372 | 0.089 | 1.561 |
|  |  |  |  | Simple mode | 0.477 | 0.798 | 0.438 | 1.454 |
|  |  |  |  | Weighted mode | 0.471 | 0.806 | 0.458 | 1.418 |
|  |  |  |  | Weighted median | 0.211 | 0.774 | 0.518 | 1.157 |
| genus.RuminococcaceaeNK4A214group(id.11358) | 16 | 0.661 | 0.219 | ivw-fe | 0.009 | 0.549 | 0.350 | 0.861 |
|  |  |  |  | MR Egger | 0.252 | 0.396 | 0.086 | 1.812 |
|  |  |  |  | Simple mode | 0.545 | 0.702 | 0.229 | 2.154 |
|  |  |  |  | Weighted mode | 0.465 | 0.684 | 0.253 | 1.849 |
|  |  |  |  | Weighted median | 0.322 | 0.712 | 0.364 | 1.394 |
| genus.Ruminococcus2(id.11374) | 15 | 0.645 | 0.571 | ivw-fe | 0.007 | 0.563 | 0.371 | 0.853 |
|  |  |  |  | MR Egger | 0.145 | 0.452 | 0.166 | 1.232 |
|  |  |  |  | Simple mode | 0.324 | 0.604 | 0.230 | 1.587 |
|  |  |  |  | Weighted mode | 0.163 | 0.559 | 0.258 | 1.212 |
|  |  |  |  | Weighted median | 0.058 | 0.564 | 0.313 | 1.019 |
| genus.unknowngenus(id.1000005479) | 10 | 0.277 | 0.694 | ivw-fe | 0.005 | 1.774 | 1.187 | 2.651 |
|  |  |  |  | MR Egger | 0.107 | 4.546 | 0.888 | 23.277 |
|  |  |  |  | Simple mode | 0.064 | 2.587 | 1.071 | 6.248 |
|  |  |  |  | Weighted mode | 0.080 | 2.194 | 1.006 | 4.782 |
|  |  |  |  | Weighted median | 0.011 | 2.014 | 1.172 | 3.462 |
| family.BacteroidalesS24.7group(id.11173) | 10 | 0.277 | 0.694 | ivw-fe | 0.005 | 1.774 | 1.187 | 2.651 |
|  |  |  |  | MR Egger | 0.107 | 4.546 | 0.888 | 23.277 |
|  |  |  |  | Simple mode | 0.049 | 2.587 | 1.142 | 5.861 |
|  |  |  |  | Weighted mode | 0.081 | 2.194 | 1.001 | 4.806 |
|  |  |  |  | Weighted median | 0.014 | 2.014 | 1.152 | 3.524 |
| family.Christensenellaceae(id.1866) | 11 | 0.514 | 0.588 | ivw-fe | 0.041 | 1.596 | 1.019 | 2.498 |
|  |  |  |  | MR Egger | 0.136 | 2.065 | 0.866 | 4.923 |
|  |  |  |  | Simple mode | 0.428 | 1.514 | 0.565 | 4.056 |
|  |  |  |  | Weighted mode | 0.396 | 1.394 | 0.669 | 2.903 |
|  |  |  |  | Weighted median | 0.129 | 1.607 | 0.871 | 2.963 |
| family.FamilyXIII(id.1957) | 11 | 0.442 | 0.785 | ivw-fe | 0.028 | 1.931 | 1.072 | 3.476 |
|  |  |  |  | MR Egger | 0.804 | 0.726 | 0.062 | 8.448 |
|  |  |  |  | Simple mode | 0.283 | 2.077 | 0.588 | 7.340 |
|  |  |  |  | Weighted mode | 0.348 | 1.860 | 0.541 | 6.394 |
|  |  |  |  | Weighted median | 0.132 | 1.814 | 0.836 | 3.934 |

No.of SNP, number of SNPs being used as IVs.; ivw-fe, fixed-effects inverse variance weighting; OR, Odds Ratio; or_lci95-or_uci95, 95% confidence interval; Significant P.value was marked in red;
