# Supplementary material for: Investigating the causal impact of gut microbiota on trigeminal neuralgia: a bidirectional Mendelian randomization study
Source: Front Microbiol. 2025 Feb 27;16:1420978. doi: 10.3389/fmicb.2025.1420978 (PMC11905160; doi:10.3389/fmicb.2025.1420978)
Supplement: Supplementary file 2 [file Table_2.docx]

**Supplementary Table2. Detailed information on SNPs.**

| Bacterial taxa | SNP | Effect allele | Other allele | F | palindromic | exposure | | | outcome | | |
| --- | --- | --- | --- | --- | --- | --- | --- | --- | --- | --- | --- |
|  |  |  |  |  |  | Beta | SE | p | Beta | SE | pval |
| genus.Butyricimonas(id.945) | rs11228830 | A | G | 21 | FALSE | 0.135 | 0.030 | 5.82E-06 | 0.083 | 0.092 | 0.368 |
|  | rs113054641 | G | A | 28 | FALSE | -0.145 | 0.027 | 1.32E-07 | -0.033 | 0.122 | 0.785 |
|  | rs12304031 | G | A | 19 | FALSE | -0.086 | 0.020 | 1.17E-05 | -0.060 | 0.077 | 0.434 |
|  | rs12458763 | A | C | 20 | FALSE | 0.122 | 0.027 | 5.99E-06 | -0.074 | 0.121 | 0.541 |
|  | rs1862649 | G | A | 21 | FALSE | 0.113 | 0.025 | 5.16E-06 | 0.073 | 0.099 | 0.459 |
|  | rs2114713 | G | T | 20 | FALSE | 0.063 | 0.014 | 6.41E-06 | 0.087 | 0.051 | 0.086 |
|  | rs2642760 | G | C | 24 | TRUE | 0.071 | 0.015 | 9.28E-07 | 0.033 | 0.055 | 0.548 |
|  | rs270727 | G | C | 21 | TRUE | -0.069 | 0.015 | 4.16E-06 | -0.051 | 0.054 | 0.347 |
|  | rs326049 | C | G | 21 | TRUE | 0.076 | 0.017 | 5.83E-06 | -0.006 | 0.057 | 0.915 |
|  | rs62130338 | G | A | 21 | FALSE | -0.073 | 0.016 | 3.71E-06 | -0.017 | 0.053 | 0.743 |
|  | rs62390301 | T | C | 25 | FALSE | -0.087 | 0.017 | 5.99E-07 | -0.081 | 0.063 | 0.197 |
|  | rs7083431 | A | C | 24 | FALSE | 0.070 | 0.014 | 1.11E-06 | -0.047 | 0.057 | 0.408 |
|  | rs71428626 | G | T | 21 | FALSE | -0.133 | 0.029 | 4.34E-06 | -0.147 | 0.142 | 0.298 |
|  | rs72814525 | A | G | 20 | FALSE | 0.066 | 0.015 | 9.22E-06 | 0.187 | 0.059 | 0.001 |
|  | rs782080 | T | A | 22 | TRUE | 0.065 | 0.014 | 2.18E-06 | -0.031 | 0.051 | 0.543 |
|  | rs78453362 | A | G | 21 | FALSE | -0.149 | 0.033 | 4.87E-06 | 0.231 | 0.161 | 0.151 |
|  | rs9657374 | C | T | 21 | FALSE | 0.068 | 0.015 | 4.35E-06 | 0.089 | 0.055 | 0.105 |
| genus.FamilyXIIIAD3011group(id.11293) | rs11126423 | C | T | 21 | FALSE | 0.090 | 0.020 | 4.08E-06 | -0.056 | 0.088 | 0.525 |
|  | rs11736617 | G | A | 19 | FALSE | -0.076 | 0.017 | 1.02E-05 | -0.017 | 0.110 | 0.875 |
|  | rs12314465 | A | G | 22 | FALSE | -0.092 | 0.020 | 3.10E-06 | -0.116 | 0.090 | 0.198 |
|  | rs12911842 | A | T | 20 | TRUE | -0.081 | 0.018 | 9.47E-06 | -0.038 | 0.090 | 0.676 |
|  | rs149302 | T | C | 20 | FALSE | -0.065 | 0.014 | 6.55E-06 | 0.024 | 0.060 | 0.684 |
|  | rs16840310 | A | G | 25 | FALSE | -0.061 | 0.012 | 6.43E-07 | -0.031 | 0.051 | 0.552 |
|  | rs16940167 | C | T | 21 | FALSE | 0.073 | 0.016 | 4.62E-06 | 0.022 | 0.064 | 0.729 |
|  | rs17156849 | G | A | 21 | FALSE | -0.113 | 0.025 | 4.18E-06 | -0.019 | 0.106 | 0.860 |
|  | rs62029761 | A | G | 22 | FALSE | 0.129 | 0.028 | 3.09E-06 | -0.038 | 0.112 | 0.738 |
|  | rs62200412 | C | T | 24 | FALSE | -0.080 | 0.016 | 1.02E-06 | -0.062 | 0.058 | 0.283 |
|  | rs72730932 | C | A | 26 | FALSE | -0.090 | 0.018 | 3.79E-07 | -0.192 | 0.086 | 0.026 |
|  | rs739451 | C | T | 19 | FALSE | 0.065 | 0.015 | 1.07E-05 | 0.049 | 0.062 | 0.433 |
|  | rs9276029 | A | G | 19 | FALSE | -0.081 | 0.019 | 1.24E-05 | -0.015 | 0.068 | 0.832 |
|  | rs9837139 | A | G | 20 | FALSE | 0.108 | 0.024 | 7.78E-06 | 0.063 | 0.091 | 0.487 |
|  | rs9852893 | C | G | 26 | TRUE | 0.066 | 0.013 | 3.45E-07 | 0.087 | 0.057 | 0.123 |
| genus.FamilyXIIIUCG001(id.11294) | rs112362903 | A | G | 20 | FALSE | -0.149 | 0.033 | 7.74E-06 | 0.005 | 0.136 | 0.970 |
|  | rs116979587 | T | A | 22 | TRUE | -0.122 | 0.026 | 3.08E-06 | 0.033 | 0.118 | 0.781 |
|  | rs12049454 | T | C | 23 | FALSE | -0.065 | 0.013 | 1.38E-06 | 0.047 | 0.052 | 0.363 |
|  | rs1426266 | T | C | 24 | FALSE | -0.067 | 0.014 | 1.22E-06 | 0.091 | 0.057 | 0.106 |
|  | rs2276529 | C | G | 21 | TRUE | -0.076 | 0.017 | 4.23E-06 | 0.082 | 0.063 | 0.190 |
|  | rs3842897 | G | A | 22 | FALSE | -0.113 | 0.024 | 3.48E-06 | 0.109 | 0.089 | 0.217 |
|  | rs62414802 | C | T | 21 | FALSE | -0.061 | 0.013 | 5.44E-06 | 0.072 | 0.058 | 0.211 |
|  | rs7119679 | G | A | 21 | FALSE | -0.081 | 0.017 | 3.67E-06 | -0.101 | 0.059 | 0.089 |
|  | rs76463770 | A | G | 21 | FALSE | 0.193 | 0.042 | 4.23E-06 | -0.158 | 0.147 | 0.282 |
|  | rs8076666 | A | G | 20 | FALSE | 0.089 | 0.020 | 7.63E-06 | -0.097 | 0.077 | 0.208 |
| genus.Lactococcus(id.1851) | rs10417872 | T | G | 23 | FALSE | 0.118 | 0.025 | 1.40E-06 | -0.025 | 0.056 | 0.653 |
|  | rs123059 | T | C | 25 | FALSE | -0.137 | 0.027 | 6.46E-07 | 0.036 | 0.061 | 0.560 |
|  | rs12621813 | G | A | 20 | FALSE | 0.108 | 0.024 | 6.24E-06 | -0.085 | 0.057 | 0.135 |
|  | rs17168302 | G | A | 20 | FALSE | 0.192 | 0.042 | 6.28E-06 | -0.049 | 0.082 | 0.548 |
|  | rs2293361 | C | T | 21 | FALSE | -0.199 | 0.043 | 3.79E-06 | 0.167 | 0.112 | 0.135 |
|  | rs34757988 | G | C | 29 | TRUE | 0.122 | 0.023 | 9.25E-08 | 0.033 | 0.052 | 0.522 |
|  | rs4766997 | C | T | 23 | FALSE | 0.115 | 0.024 | 1.53E-06 | -0.001 | 0.051 | 0.984 |
|  | rs55910161 | C | T | 23 | FALSE | 0.146 | 0.031 | 1.90E-06 | -0.170 | 0.080 | 0.034 |
|  | rs6674304 | C | T | 21 | FALSE | 0.201 | 0.044 | 5.60E-06 | -0.151 | 0.132 | 0.254 |
|  | rs757872 | G | C | 26 | TRUE | 0.141 | 0.028 | 3.31E-07 | -0.033 | 0.067 | 0.624 |
|  | rs7992246 | T | C | 20 | FALSE | 0.104 | 0.023 | 6.29E-06 | -0.029 | 0.052 | 0.573 |
| genus.RuminococcaceaeNK4A214group(id.11358) | rs11241747 | C | T | 20 | FALSE | 0.053 | 0.012 | 8.69E-06 | -0.058 | 0.055 | 0.298 |
|  | rs114244418 | C | G | 22 | TRUE | -0.175 | 0.037 | 2.58E-06 | 0.060 | 0.128 | 0.641 |
|  | rs11586410 | G | A | 26 | FALSE | -0.086 | 0.017 | 3.76E-07 | 0.143 | 0.070 | 0.041 |
|  | rs12642039 | T | C | 21 | FALSE | -0.055 | 0.012 | 3.63E-06 | 0.051 | 0.052 | 0.334 |
|  | rs12731 | A | G | 21 | FALSE | -0.053 | 0.012 | 4.51E-06 | 0.087 | 0.052 | 0.094 |
|  | rs13087692 | T | G | 21 | FALSE | 0.057 | 0.013 | 5.05E-06 | 0.101 | 0.055 | 0.065 |
|  | rs136761 | G | A | 24 | FALSE | -0.059 | 0.012 | 8.19E-07 | -0.054 | 0.052 | 0.300 |
|  | rs147475196 | A | G | 21 | FALSE | -0.134 | 0.030 | 5.86E-06 | 0.045 | 0.082 | 0.581 |
|  | rs34576931 | G | C | 20 | TRUE | -0.087 | 0.019 | 7.42E-06 | 0.022 | 0.099 | 0.823 |
|  | rs35559912 | T | C | 21 | FALSE | -0.093 | 0.020 | 5.57E-06 | 0.220 | 0.077 | 0.004 |
|  | rs4814689 | C | T | 22 | FALSE | -0.108 | 0.023 | 2.68E-06 | 0.180 | 0.122 | 0.140 |
|  | rs5994253 | A | G | 26 | FALSE | -0.081 | 0.016 | 2.64E-07 | 0.015 | 0.072 | 0.839 |
|  | rs62027366 | T | C | 20 | FALSE | 0.062 | 0.014 | 7.75E-06 | -0.037 | 0.063 | 0.555 |
|  | rs6681678 | C | T | 17 | FALSE | -0.100 | 0.024 | 2.99E-05 | -0.007 | 0.141 | 0.963 |
|  | rs73158814 | C | G | 23 | TRUE | -0.109 | 0.023 | 1.56E-06 | -0.004 | 0.135 | 0.974 |
|  | rs7573569 | T | C | 21 | FALSE | 0.108 | 0.023 | 4.00E-06 | -0.102 | 0.105 | 0.328 |
| genus.Ruminococcus2(id.11374) | rs10199274 | G | C | 23 | TRUE | 0.066 | 0.014 | 1.94E-06 | -0.011 | 0.064 | 0.859 |
|  | rs12406309 | A | C | 20 | FALSE | -0.063 | 0.014 | 8.93E-06 | 0.015 | 0.061 | 0.810 |
|  | rs1819812 | G | T | 21 | FALSE | 0.084 | 0.018 | 5.22E-06 | 0.037 | 0.113 | 0.745 |
|  | rs2368224 | T | G | 21 | FALSE | 0.200 | 0.044 | 5.28E-06 | -0.071 | 0.116 | 0.543 |
|  | rs2846589 | G | T | 20 | FALSE | 0.052 | 0.012 | 7.26E-06 | -0.041 | 0.051 | 0.430 |
|  | rs2997412 | A | G | 22 | FALSE | -0.057 | 0.012 | 3.37E-06 | 0.061 | 0.056 | 0.281 |
|  | rs4400279 | A | G | 21 | FALSE | 0.055 | 0.012 | 5.42E-06 | 0.003 | 0.054 | 0.953 |
|  | rs4799823 | C | T | 21 | FALSE | 0.084 | 0.018 | 4.38E-06 | -0.058 | 0.066 | 0.378 |
|  | rs55707116 | C | A | 21 | FALSE | 0.087 | 0.019 | 4.74E-06 | -0.127 | 0.096 | 0.184 |
|  | rs58681734 | A | G | 20 | FALSE | 0.072 | 0.016 | 6.73E-06 | 0.056 | 0.062 | 0.369 |
|  | rs61791565 | T | C | 20 | FALSE | -0.052 | 0.012 | 7.83E-06 | -0.041 | 0.051 | 0.419 |
|  | rs75140805 | T | G | 22 | FALSE | 0.084 | 0.018 | 2.11E-06 | -0.041 | 0.067 | 0.535 |
|  | rs7635831 | G | A | 23 | FALSE | 0.062 | 0.013 | 1.65E-06 | -0.146 | 0.053 | 0.006 |
|  | rs7693984 | G | A | 19 | FALSE | -0.103 | 0.024 | 1.22E-05 | 0.174 | 0.122 | 0.152 |
|  | rs78120384 | A | G | 24 | FALSE | -0.193 | 0.039 | 8.73E-07 | 0.158 | 0.088 | 0.071 |
| genus.unknowngenus(id.1000005479) | rs10872669 | A | G | 20 | FALSE | -0.123 | 0.028 | 8.03E-06 | -0.155 | 0.089 | 0.080 |
|  | rs11135366 | C | G | 21 | TRUE | 0.084 | 0.018 | 4.58E-06 | -0.003 | 0.059 | 0.965 |
|  | rs12748533 | G | T | 23 | FALSE | -0.082 | 0.017 | 1.99E-06 | 0.044 | 0.056 | 0.430 |
|  | rs17043770 | G | A | 26 | FALSE | -0.177 | 0.034 | 2.74E-07 | -0.122 | 0.082 | 0.137 |
|  | rs61508842 | T | C | 20 | FALSE | 0.123 | 0.027 | 6.80E-06 | 0.139 | 0.089 | 0.118 |
|  | rs6831034 | T | A | 22 | TRUE | -0.096 | 0.021 | 3.15E-06 | -0.117 | 0.088 | 0.183 |
|  | rs689695 | C | A | 24 | FALSE | 0.081 | 0.017 | 1.08E-06 | 0.010 | 0.055 | 0.860 |
|  | rs738193 | T | C | 26 | FALSE | 0.085 | 0.017 | 3.24E-07 | 0.074 | 0.053 | 0.158 |
|  | rs78609301 | A | G | 20 | FALSE | -0.087 | 0.020 | 9.41E-06 | -0.030 | 0.055 | 0.580 |
|  | rs941000 | C | T | 27 | FALSE | 0.085 | 0.016 | 1.95E-07 | 0.071 | 0.052 | 0.176 |
| family.BacteroidalesS24.7group(id.11173) | rs10872669 | A | G | 20 | FALSE | -0.123 | 0.028 | 8.03E-06 | -0.155 | 0.089 | 0.080 |
|  | rs11135366 | C | G | 21 | TRUE | 0.084 | 0.018 | 4.58E-06 | -0.003 | 0.059 | 0.965 |
|  | rs12748533 | G | T | 23 | FALSE | -0.082 | 0.017 | 1.99E-06 | 0.044 | 0.056 | 0.430 |
|  | rs17043770 | G | A | 26 | FALSE | -0.177 | 0.034 | 2.74E-07 | -0.122 | 0.082 | 0.137 |
|  | rs61508842 | T | C | 20 | FALSE | 0.123 | 0.027 | 6.80E-06 | 0.139 | 0.089 | 0.118 |
|  | rs6831034 | T | A | 22 | TRUE | -0.096 | 0.021 | 3.15E-06 | -0.117 | 0.088 | 0.183 |
|  | rs689695 | C | A | 24 | FALSE | 0.081 | 0.017 | 1.08E-06 | 0.010 | 0.055 | 0.860 |
|  | rs738193 | T | C | 26 | FALSE | 0.085 | 0.017 | 3.24E-07 | 0.074 | 0.053 | 0.158 |
|  | rs78609301 | A | G | 20 | FALSE | -0.087 | 0.020 | 9.41E-06 | -0.030 | 0.055 | 0.580 |
|  | rs941000 | C | T | 27 | FALSE | 0.085 | 0.016 | 1.95E-07 | 0.071 | 0.052 | 0.176 |
| family.Christensenellaceae(id.1866) | rs117186816 | G | A | 22 | FALSE | -0.206 | 0.044 | 2.23E-06 | -0.036 | 0.088 | 0.682 |
|  | rs12380890 | A | G | 21 | FALSE | -0.050 | 0.011 | 5.67E-06 | 0.035 | 0.050 | 0.492 |
|  | rs12657403 | A | G | 21 | FALSE | 0.078 | 0.017 | 5.52E-06 | 0.041 | 0.114 | 0.720 |
|  | rs4076564 | G | A | 23 | FALSE | -0.189 | 0.039 | 1.50E-06 | -0.109 | 0.112 | 0.330 |
|  | rs4805124 | C | G | 20 | TRUE | 0.049 | 0.011 | 8.17E-06 | -0.028 | 0.050 | 0.580 |
|  | rs5763231 | C | T | 21 | FALSE | -0.068 | 0.015 | 5.06E-06 | -0.039 | 0.058 | 0.502 |
|  | rs62573205 | G | A | 24 | FALSE | -0.065 | 0.013 | 9.35E-07 | -0.099 | 0.063 | 0.116 |
|  | rs7211194 | C | T | 20 | FALSE | 0.049 | 0.011 | 9.14E-06 | 0.005 | 0.050 | 0.929 |
|  | rs72706624 | G | T | 20 | FALSE | 0.088 | 0.020 | 7.92E-06 | 0.189 | 0.082 | 0.021 |
|  | rs77867022 | G | T | 18 | FALSE | -0.160 | 0.038 | 2.37E-05 | -0.135 | 0.097 | 0.161 |
|  | rs870002 | C | T | 20 | FALSE | 0.049 | 0.011 | 8.49E-06 | -0.040 | 0.051 | 0.438 |
|  | rs892686 | A | G | 21 | FALSE | 0.051 | 0.011 | 3.92E-06 | 0.009 | 0.050 | 0.866 |
| family.FamilyXIII(id.1957) | rs10404377 | C | A | 20 | FALSE | -0.050 | 0.011 | 6.58E-06 | 0.043 | 0.052 | 0.409 |
|  | rs118170811 | A | G | 23 | FALSE | 0.152 | 0.032 | 1.74E-06 | -0.013 | 0.145 | 0.928 |
|  | rs12643275 | A | T | 22 | TRUE | -0.055 | 0.012 | 2.25E-06 | -0.071 | 0.055 | 0.194 |
|  | rs12904405 | A | G | 20 | FALSE | -0.085 | 0.019 | 9.82E-06 | -0.038 | 0.086 | 0.662 |
|  | rs1999289 | A | T | 21 | TRUE | -0.064 | 0.014 | 4.73E-06 | -0.019 | 0.060 | 0.754 |
|  | rs3098182 | G | T | 21 | FALSE | 0.051 | 0.011 | 3.89E-06 | 0.056 | 0.050 | 0.268 |
|  | rs482905 | G | T | 22 | FALSE | 0.060 | 0.013 | 2.72E-06 | 0.038 | 0.058 | 0.518 |
|  | rs6501525 | A | G | 24 | FALSE | 0.056 | 0.012 | 1.18E-06 | 0.120 | 0.053 | 0.024 |
|  | rs66753613 | G | A | 20 | FALSE | 0.065 | 0.014 | 6.36E-06 | 0.065 | 0.065 | 0.321 |
|  | rs6797051 | C | T | 22 | FALSE | -0.081 | 0.017 | 2.56E-06 | -0.016 | 0.085 | 0.849 |
|  | rs7076829 | T | A | 21 | TRUE | -0.052 | 0.011 | 4.27E-06 | 0.111 | 0.052 | 0.034 |
|  | rs7514702 | T | C | 22 | FALSE | -0.066 | 0.014 | 2.83E-06 | -0.061 | 0.070 | 0.387 |

SNP, single nucleotide polymorphism; SE, Standard error;
