# Supplementary figures and images for: Investigating the causal impact of gut microbiota on trigeminal neuralgia: a bidirectional Mendelian randomization study
Source: Front Microbiol. 2025 Feb 27;16:1420978. doi: 10.3389/fmicb.2025.1420978 (PMC11905160; doi:10.3389/fmicb.2025.1420978)

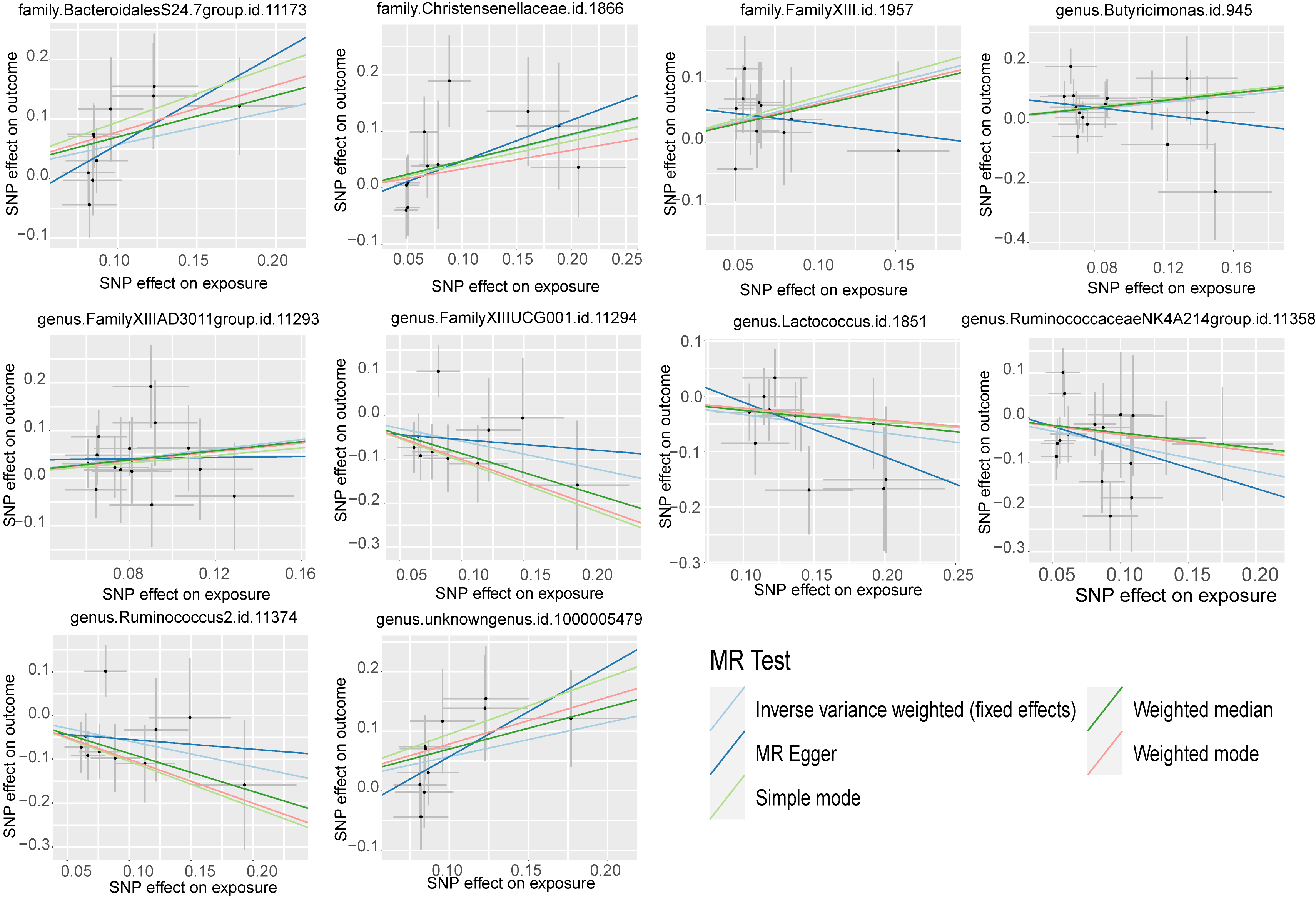

Supplement: Supplementary file 3 [file Image_1.tif]
